# Guillain-Barré Syndrome and Visual Impairment Associated with Emerging Oropouche Virus Lineage, Brazil, 2024

**DOI:** 10.3201/eid3204.250617

**Published:** 2026-04

**Authors:** Carlos Garcia Filho, Fernanda Martins Maia Carvalho, Antonio Silva Lima Neto, Ana Maria Cabral Maia, Matheus Andrighetti Rossi, Milena Sales Pitombeira, Paula Camila Alves de Assis Pereira Matos, Tania Mara Silva Coelho, Lauro Vieira Perdigão Neto, Lívia Mendes de Almeida, Felipe Gomes Naveca, Carla Santos de Oliveira, Fernanda de Bruycker-Nogueira, Ana Maria Bispo de Filippis, Kleber Giovanni Luz, André Ricardo Ribas Freitas, Luciano Pamplona de Góes Cavalcanti

**Affiliations:** Secretaria da Saúde do Estado do Ceará, Fortaleza, Brazil (C. Garcia Filho, A.S. Lima Neto, A.M.C. Maia, T.M.S. Coelho, L.V.P. Neto); Universidade de Fortaleza, Fortaleza (C. Garcia Filho, F.M.M. Carvalho, A.S. Lima Neto); Hospital Geral de Fortaleza, Fortaleza (F.M.M. Carvalho, M.A. Rossi, M.S. Pitombeira, P.C.A. de Assis Pereira Matos); Programa de Pós-graduação em Saúde Pública da Universidade Federal do Ceará, Fortaleza (A.M.C. Maia); Universidade Federal do Ceará, Fortaleza (L.V.P. Neto); Faculdade de Medicina da Universidade de São Paulo, São Paulo, Brazil (L.V.P. Neto); Centro Universitário Christus, Fortaleza (L.M. de Almeida, L.P. de Góes Cavalcanti); Programa de Pós-graduação em Patologia da Universidade Federal do Ceará, Fortaleza (L.M. de Almeida, L.P. de Góes Cavalcanti); Instituto Oswaldo Cruz, Rio de Janeiro, Brazil (F.G. Naveca, C.S. de Oliveira, F. de Bruycker-Nogueira, A.M.B. de Filippis); Universidade Federal do Rio Grande do Norte, Natal, Brazil (K.G. Luz); São Leopoldo Mandic, Campinas, Brazil (A.R.R. Freitas); Escola de Saúde Pública do Ceará, Fortaleza (L.P. de Góes Cavalcanti)

**Keywords:** Oropouche virus, Guillain-Barré syndrome, vector-borne infections, viruses, emerging communicable diseases, Arbovirus, Brazil

## Abstract

We report a case of Guillain-Barré syndrome with visual impairment after confirmed Oropouche virus infection during the 2024 outbreak in Ceará, Brazil. Whole-genome sequencing revealed infection by a novel reassortant viral lineage (OROVBR_2025_2024), raising concern about the neurovirulence of this emerging orthobunyavirus strain.

Oropouche virus (OROV), an orthobunyavirus transmitted by mosquitoes and *Culicoides paraensis* midges, is emerging as a major arboviral pathogen in Latin America. Although typically associated with mild febrile illness, the current outbreak, which is linked to the novel reassortant lineage OROV_BR-2015–2024 _(*1*), has been associated with severe cases, including fatalities, vertical transmission with fetal deaths (*2*–*4*), and Guillain-Barré syndrome (GBS) (*5*). We report a case of GBS with possible bilateral optic neuritis after OROV infection during the 2024 outbreak in Ceará state, Brazil.

## The Study

On August 12, 2024, a previously healthy 48-year-old woman from Capistrano in Ceará state developed acute febrile illness, characterized by high fever (39°C), chills, severe headache, and myalgia. OROV cases had been previously documented in this locality (*2*,*6*). On August 14, OROV infection was confirmed through quantitative reverse transcription PCR (qRT-PCR) performed by the Ceará Central Public Health Laboratory. Dengue, Zika, chikungunya, and Mayaro virus infections were excluded through both molecular and serologic testing (Table) (*7*,*8*). Fever and systemic symptoms persisted for 2 weeks and were followed by the onset of neurologic manifestations, including paresthesia in her lower limbs, which progressively developed into ascending paresis affecting both upper and lower limbs. In addition, bilateral facial weakness and profound visual loss developed. Initial treatment with corticosteroids (prednisone 40 mg/day for 7 days) was prescribed on September 15 but improvement was minimal, and the patient was referred to a tertiary hospital.

**Table:** Results of laboratory screening in study of Guillain-Barré syndrome and visual impairment associated with emerging Oropouche virus lineage, Brazil, 2024*

Laboratory test	Result	Reference range or detection method
Hematology and biochemistry		
Hemoglobin	15.3 g/dL	11.5–15.0 g/dL
Leukocytes	8,500/µL	3,600–11,000 cells/µL
Platelets	346,000/µL	150,000–450,000/µL
C-reactive protein	5 mg/L	<5 mg/L
Erythrocyte sedimentation rate	27 mm/h	0–20 mm/h
Calcium	10.7 mg/dL	8.50–10.50 mg/dL
Creatinine	0.72 mg/dL	0.60–1.10 mg/dL
Alanine aminotransferase	63 U/L	<31 U/L
Aspartate aminotransferase	22 U/L	<32 U/L
C3	118 mg/dL	90–180 mg/dL
C4	43.8 mg/dL	10–40 mg/dL
Rheumatoid factor	20.7 IU/mL	0–15 IU/mL
Cerebrospinal fluid analysis		
Cell count	2 cells/µL	0–4 cells/µL
Protein	156 mg/dL	15–40 mg/dL
Glucose	135 mg/dL	40–70 mg/dL
Adenosine deaminase	0.72 U/L	0–9 U/L
Infectious disease markers		
HIV	Nonreactive	Chemiluminescent immunoassay
Hepatitis B surface antigen	Nonreactive	Chemiluminescent immunoassay
Hepatitis C	Nonreactive	Chemiluminescent immunoassay
Syphilis/neurosyphilis	Nonreactive	Flocculation (VDRL)
*Mycobacterium tuberculosis* DNA	Nonreactive	Xpert MTB/RIF (Cepheid, https://www.cepheid.com)
Zika virus†	Nonreactive	qRT-PCR, ELISA IgM (serum and CSF)
Chikungunya virus†	Nonreactive	qRT-PCR, ELISA IgM (serum and CSF)
Dengue virus†	Nonreactive	qRT-PCR, ELISA IgM (serum and CSF
Oropouche virus†	Positive	qRT-PCR (serum)
Autoimmune profile		
Antinuclear antibodies‡	Negative	Indirect immunofluorescence assay on Hep-2 cells
Aquaporin-4 antibodies‡	Negative	Cell-based assay
MOG antibodies‡	Negative	Cell-based assay

*MOG, myelin oligodendrocyte glycoprotein; qRT-PCR, quantitative reverse transcription PCR; VDRL, Venereal Disease Research Laboratory. †Oropouche virus qRT-PCR and qRT-PCR for dengue, Zika, chikungunya, and Mayaro viruses, as well as serologic testing, were performed using serum collected on August 14, 2024. ‡Autoimmune screening tests, including antinuclear antibody (ANA), and aquaporin 4 and MOG antibodies, were collected during the home visit on February 5, 2025. All other tests were conducted during hospitalization, September 17–October 1, 2024.

Upon admission on September 17, 2024, the patient was experiencing flaccid tetraparesis, bilateral facial paresis, and areflexia. Blood tests results were unremarkable. Analysis of cerebrospinal fluid revealed albuminocytologic dissociation, elevated protein levels, and unremarkable cell counts (Table), supporting a diagnosis of peripheral demyelinating neuropathy. Oligoclonal bands and IgG index testing of cerebrospinal fluid were unavailable.

Electroneuromyography showed absent sensory conduction in the left superficial fibular nerve, mildly prolonged motor latencies, and reduced motor potentials in fibular and tibial nerves. F-waves were delayed or absent, whereas the H-reflex was bilaterally absent. Electroneuromyography revealed reduced recruitment with high motor unit activity during voluntary movement, compatible with peripheral nervous system demyelination (Appendix Tables 1–5).

**Figure 1:**
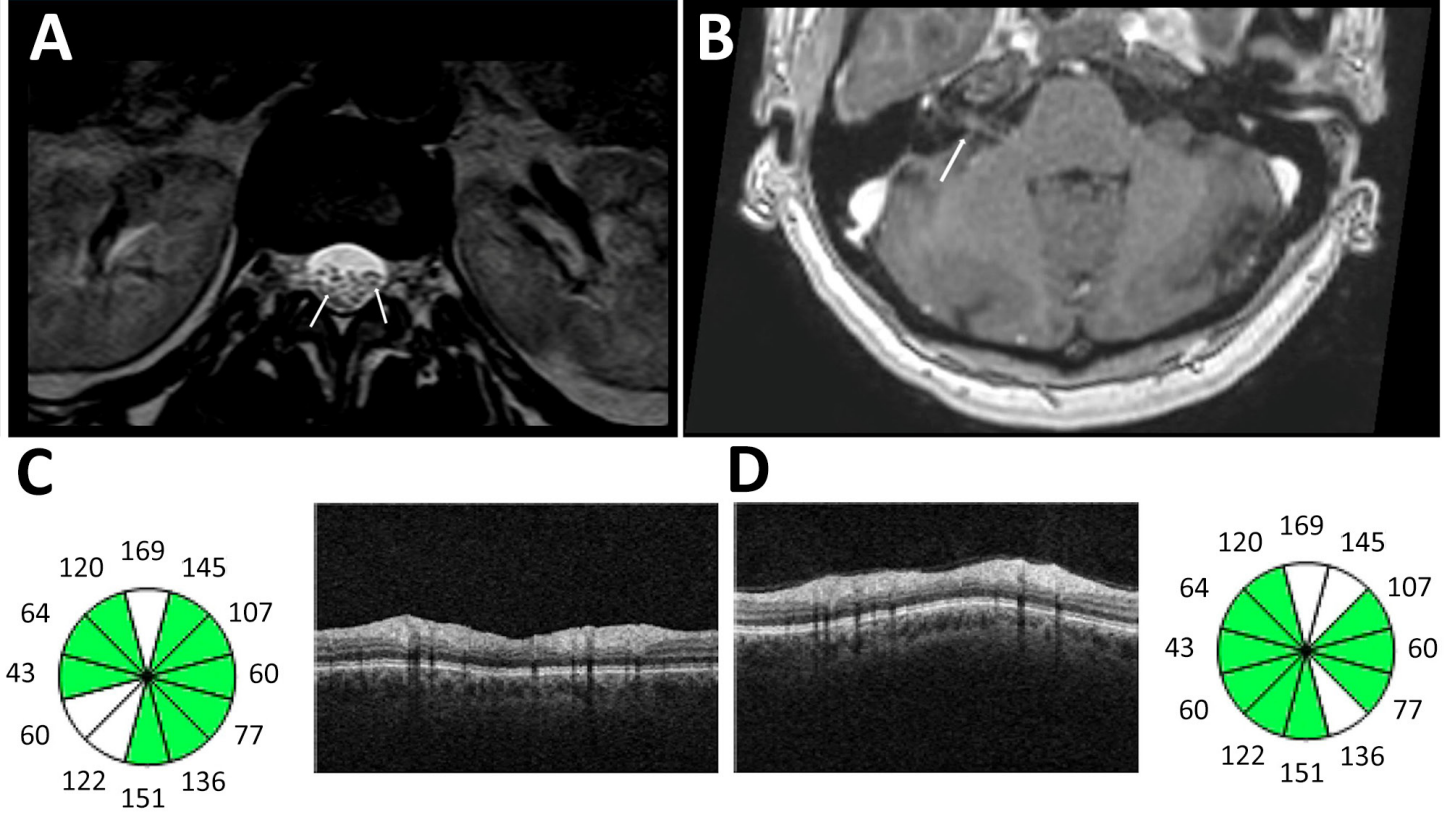
Imaging from study of Guillain-Barré syndrome and visual impairment associated with emerging Oropouche virus lineage, Brazil, 2024. A) T2-weighted magnetic resonance imaging of lumbar spinal cord. Arrows indicate nerve root edema. B) T1-weighted magnetic resonance imaging of brain with contrast. Arrow indicates enhancement of right facial nerve. C, D) Optical coherence tomography of right eye (C) and left eye (D) revealed bilateral loss of ganglionic cells in the papillomacular bundle, shown in white on the circle graphic.

Magnetic resonance imaging (MRI) of the brain showed bilateral facial nerve swelling; cervical, thoracic, and lumbar spine MRI revealed edema of lumbar nerve roots (Figure 1). Orbital MRI results were unremarkable.

Bilateral vision loss developed, accompanied by painful eye movements, compromising functional capacity as reported by the patient and family. Although the neuroophthalmologic evaluation occurred only 3 weeks after onset and during steroid treatment, the examination revealed corrected visual acuity of 20/50 in both eyes, impaired pupillary light reflexes, red dyschromatopsia, nasal optic disc edema on fundoscopy, and peripapillary retinal nerve fiber layer edema on optical coherence tomography (Figure 1) without macular involvement. Visual field testing was not performed because of technical issues, and results of MRI performed at this stage were unremarkable. The evidence of subacute vision loss associated with orbital pain worsening with eye movement, reduced contrast and color vision, and pupillary deficit along with the mild optic disc swelling seen in optical coherence tomography favors the diagnosis of a possible optic neuritis (*9*). The temporal association of those findings with a preceding infectious episode (2–4 weeks) supports the possibility of bilateral postinfectious optic neuritis captured later in the disease evolution.

Empiric antiviral drugs and antibiotics were initiated but discontinued after excluding infectious etiologies such as *Listeria* and herpes. Plasmapheresis was administered in 5 sessions and was generally well tolerated, although 1 session was followed by transient dysautonomia.

The patient showed gradual but partial neurologic improvement and was discharged on October 1 with moderate gains in motor function and visual acuity. At the 90-day follow-up, the patient was able to walk with assistance, and her visual acuity remained at 20/50 in both eyes. Mild proprioceptive and sensory deficits persisted, particularly in the left foot. Deep tendon reflexes were normal except for slightly diminished Achilles reflexes.

A home visit on February 5, 2025 (6 months after symptom onset), revealed that the patient continued to experience persistent visual impairment, distal hypoesthesia, and paresis in the right lower limb, as well as difficulty standing for >15 minutes. She remained unable to resume agricultural activities or manage household routines independently and required assistance from family members for daily living. Despite partial neurologic improvement, those ongoing limitations led to functional dependence and the need for social security support. During this home visit, additional autoimmune screening tests, including tests for antinuclear antibody and aquaporin 4 and myelin oligodendrocyte glycoprotein antibodies, were performed. Results of all tests were negative (Table).

We performed whole-genome sequencing on patient OROV samples using an amplicon-based protocol (*7*). We recovered and concatenated near-complete coding sequences of all 3 genome segments (large, medium, and small) for phylogenetic reconstruction using maximum-likelihood inference on IQ-TREE multicore version 2.1.1 (*10*), employing a nonredundant dataset containing previously published sequences (*1*,*2*). Sequence data are available on GISAID (https://www.gisaid.org; accession no. EPI_ISL_20332357).

**Figure 2:**
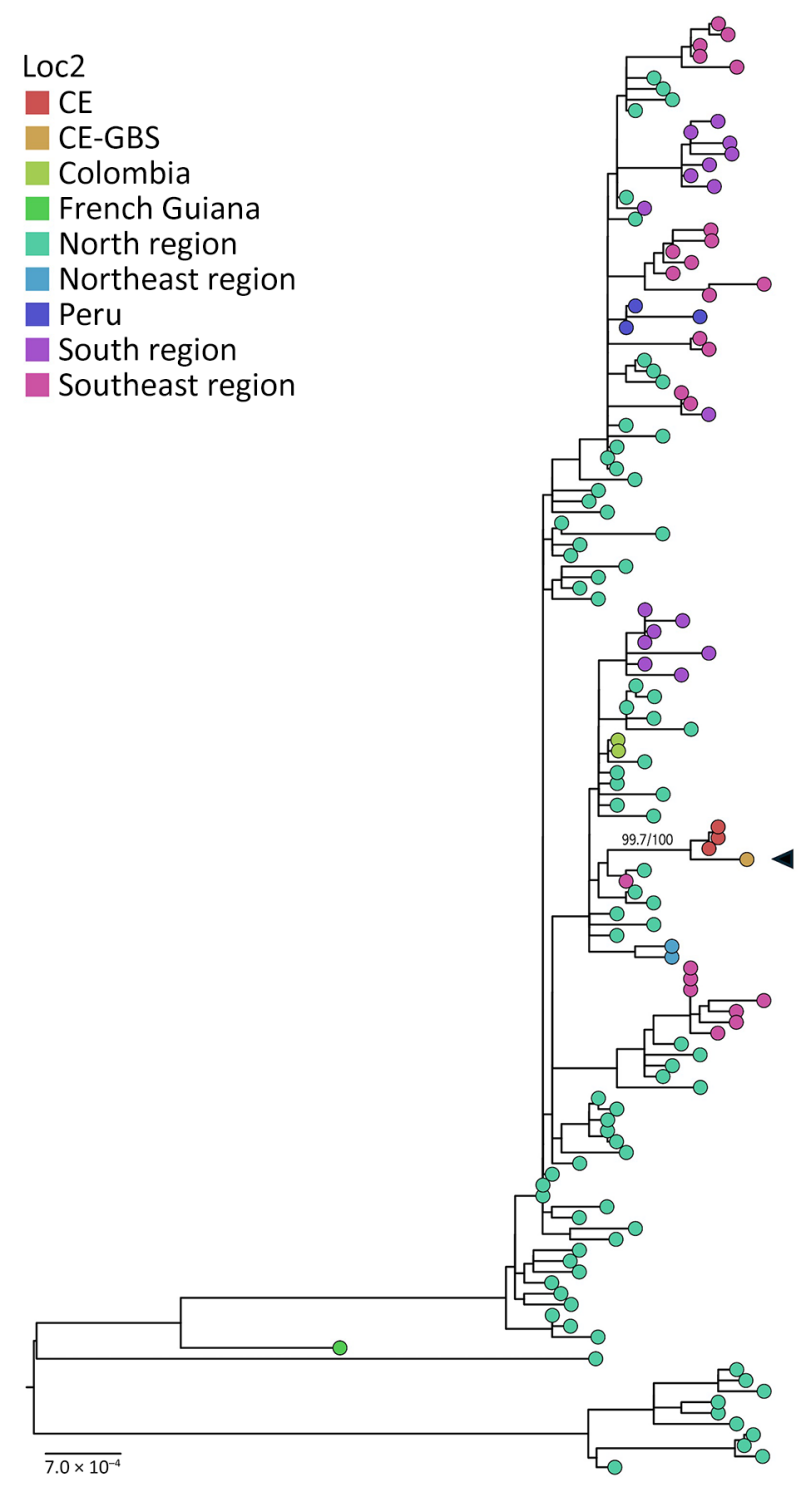
Maximum-likelihood tree of Oropouche virus concatenated segments from study of Guillain-Barré syndrome and visual impairment associated with emerging Oropouche virus lineage, Brazil, 2024. Black arrow indicates sequence from study patient. A reference dataset containing concatenated segments (large, medium, and small) of the recent Oropouche virus outbreak (2022–2024) representing different regions in Brazil and sequences obtained from Peru and Colombia were aligned using MAFFT version 7.490 (https://mafft.cbrc.jp/alignment/software/source.html) embedded in Geneious Prime 2025.0.3 (https://www.geneious.com). Subsequently, we used that alignment for phylogenomic reconstruction by maximum-likelihood using IQ-TREE multicore version 2.1.1 COVIDedition for Mac OS X 64-bit (http://www.iqtree.org). MODEL-FINDER (https://iqtree.github.io/ModelFinder) was used for evolutionary model choice, and 2,000 ultra-fast bootstraps and 2,000 SH-aLRT replicates were run to access the branches’ support (support for the CE clade is shown). The maximum-likelihood tree was edited with FigTree version 1.4.4 (https://tree.bio.ed.ac.uk/software/figtree). Scale bar indicates nucleotide substitutions per site. CE, Ceará state; CE-GBS, Ceará–Guillain-Barré syndrome.

The genome generated belongs to the new OROV_BR-2023–2024 _lineage (*1*,*11*). Phylogenetic analysis (Figure 2) revealed a highly supported monophyletic clade (UltraFast Bootstrap 99.5, SH-aLRT 100) containing sequences from other severe cases, including a fatal vertical transmission case previously described in Ceará (*2*). This clade is part of a broader lineage linked to cases in Santa Catarina, Paraná, Amazonas, and Pernambuco states in Brazil, as well as to cases in Leticia, Colombia. Those sequences belong to the previously described AM-I sublineage of OROV_BR-2015–2024 _(*1*,*2*,*11*).

## Conclusions

We report a case of GBS with visual impairment possibly caused by optic neuritis associated with the novel OROV_BR-2015–2024_ lineage, supported by clinical, laboratory, neuroimaging, electrophysiological, and genomic findings. Of note, the patient had been previously healthy and had no underlying conditions.

This case represents the second unusual severe event reported in this small region of Ceará, despite only a few hundred confirmed OROV cases, one of which involved a fetal death attributed to vertical transmission (*2*,*6*). This case highlights the neuropathogenic potential of OROV to trigger severe conditions. Although the patient received corticosteroids, intravenous immunoglobulin, and plasmapheresis, she continues to have severe neurologic impairment; whether that persistence is a result of delayed therapy or reflects a severe manifestation of OROV-associated GBS remains unclear. The phylogenetic relationship between this case and previously described cases of fetal and adult deaths (*2*,*4*), as well as GBS clusters reported in Cuba (*5*), reinforces the hypothesis that this viral lineage might possess increased pathogenicity (*12*).

Appendix Additional information about Guillain-Barré syndrome and visual impairment associated with emerging Oropouche virus lineage, Brazil, 2024.
