# Supplementary material for: Guillain-Barré Syndrome and Visual Impairment Associated with Emerging Oropouche Virus Lineage, Brazil, 2024
Source: Emerg Infect Dis. 2026 Apr;32(4):644–8. doi: 10.3201/eid3204.250617 (PMC13094853; doi:10.3201/eid3204.250617)
Supplement: Appendix — Additional information about Guillain-Barré syndrome and visual impairment associated with emerging Oropouche virus lineage, Brazil, 2024. [file 25-0617-Techapp-s1.pdf]

Article DOI: https://doi.org/10.3201/eid3204.250617

EID cannot ensure accessibility for supplementary materials supplied by authors. Readers who have difficulty accessing supplementary content should contact the authors for assistance.

Guillain-Barré Syndrome and Visual Impairment Associated with Emerging Oropouche Virus Lineage, Brazil, 2024

Appendix

Supplementary Data

Neurophysiology Laboratory Values and Peripheral Nerve Study Details

Appendix Table 1. Motor Nerve Conduction*

Rec Site	P-T Amp (mV)	Onset (ms)	Full Dur (ms)	Full Area (mVms)	Site1	Site2	Delta-0 (ms)	Dist (mm)	Vel (m/s)
Right Facial – Orbicular Eye R Motor (m. orbicular eye)	0.7	5.5	13.36	4.16	mastoid	m. orbicular eye	5.5	80	—
Left Facial – Orbicular Eye L Motor (m. orbicular eye)	1.2	4.8	9.92	3.76	mastoid	m. orbicular eye	4.8	80	—
Left Common Fibular Motor (m. EDB)	1.8	6.1	13.52	7.18	ankle	m. EDB	6.1	80	—
	0.9	13.1	22.19	5.00	below fibular head	ankle	7.0	315	45
	0.8	14.9	25.63	4.47	above fibular head	below fibular head	1.8	90	50
Right Common Fibular Motor (m. EDB)	4.3	5.7	13.83	17.84	ankle	m. EDB	5.7	80	—
	2.5#	12.6	14.77	11.77	below fibular head	ankle	6.5	310	45
	2.7	14.8	16.48	14.62	above fibular head	below fibular head	2.6	100	45
Left Deep Fibular Motor (m. tibialis anterior)	5.2	4.5	20.23	33.73	above fibular head	m. tibialis anterior	6.2	0	—
	4.5	6.2	21.17	31.64	below fibular head	Below fibular head	1.7	90	53
Right Deep Fibular Motor (m. tibialis anterior)	5.5	3.7	19.53	34.56	below fibular head	m. tibialis anterior	6.2	110	—
	5.2	6.2	20.70	33.11	above fibular head	below fibular head	2.5	90	36
Left Median Motor (m. APB)	3.0	10.5	23.36	19.31	wrist	m. APB	10.5	70	—
	2.5	15.0	19.69	14.47	elbow	wrist	4.5	215	48
	2.5	17.5	19.69	14.43	axilla	elbow	2.5	130	52
Right Median Motor (m. APB)	3.3	10.9	19.30	19.09	wrist	m. APB	10.9	70	—
	2.9	15.9	19.61	22.00	elbow	wrist	5.0	210	42
	3.0	18.3	19.45	19.45	axilla	elbow	2.4	100	42
Left Tibial Motor (m. abd hallucis)	3.5	4.7	14.06	17.51	ankle	m. abd hallucis	4.7	100	—

Rec Site	P-T Amp (mV)	Onset (ms)	Full Dur (ms)	Full Area (mVms)	Site1	Site2	Delta-0 (ms)	Dist (mm)	Vel (m/s)
	2.8	13.6	17.97	15.51	popliteal fossa	ankle	2.4	370	42
Right Tibial Motor (m. abd hallucis)	3.6	7.3	17.66	18.30	ankle	m. abd hallucis	7.3	100	—
	3.1	16.3	20.47	15.21	popliteal fossa	ankle	9.0	415	46
Left Ulnar Motor (m. abd 5th finger)	5.5	4.6	14.22	23.80	wrist	m. abd 5th finger	4.6	70	—
	4.6	9.6	15.86	22.58	below elbow	wrist	5.0	185	37
	4.6	11.2	16.88	23.59	above elbow	below elbow	1.6	100	63
	3.1	13.0	15.63	15.19	axilla	above elbow	1.8	130	72
Right Ulnar Motor (m. abd 5th finger)	5.4	3.3	16.72	26.38	wrist	m. abd 5th finger	3.3	70	—
	4.5	8.9	15.08	19.10	below elbow	wrist	5.6	200	36
	3.9	10.9	18.36	20.39	above elbow	below elbow	2.0	100	50
	3.4	12.7	18.13	18.35	axilla	above elbow	1.8	90	50

*PAMC (Pattern of Abnormal Motor Conduction) of mildly to moderately decreased amplitudes in ulnar, tibial, and right common fibular nerves (EDB) and deep fibular nerves (m. TA); moderately decreased in median nerves, tibials, and left common fibular nerve (m. EDB), associated with markedly increased motor latencies in median nerves and normal in other nerves.
Motor conduction velocity (MCV) is mildly decreased in the right median and left tibial nerves, and moderately decreased in the ulnar nerves (elbow–wrist segment), moderately decreased in deep fibular nerves (m. TA), and normal in the remaining nerves studied.
Partial motor conduction block around 42–25% in the fibular nerves (recorded at m. EDB), with temporal dispersion in the left fibular nerve.
Facial nerves show mildly (left) to moderately (right) reduced motor amplitudes, and motor latencies are normal on the right and slightly increased on the left.

Appendix Table 2. Sensory Nerve Action Potential

Rec Site	P-T Amp (µV)	Onset (ms)	Neg Dur (ms)	Site1	Site2	Dist (mm)	Vel (m/s)
Left Superficial Fibular Sensory Antidromic (foot dorsum)	16.7	1.7	1.63	anterior ankle	foot dorsum	90	53
Right Superficial Fibular Sensory Antidromic (foot dorsum)	9.4	2.4	1.41	anterior ankle	foot dorsum	95	40
Left Median Sensory Antidromic (2nd finger)	-	—	0.16	wrist	2nd finger	140	—
Right Median Sensory Antidromic (2nd finger)	-	—	—	wrist	2nd finger	140	—
Left Sural Sensory Antidromic (lateral malleolus)	25.2	1.4	1.50	lateral leg surface	lateral malleolus	80	57
Right Sural Sensory Antidromic (lateral malleolus)	18.1	1.4	1.41	lateral leg surface	lateral malleolus	80	57
Left Ulnar Sensory Antidromic (5th finger)	—	—	—	wrist	5th finger	120	—
Right Ulnar Sensory Antidromic (5th finger)	—	—	13.72	wrist	5th finger	115	—

SNAPs (sensory nerve action potentials) indeterminate in median (1st and 2nd fingers) and ulnar (5th finger) nerves bilaterally.
SNAP amplitudes are mildly decreased in the right superficial fibular and radial sensory nerves.
SNAP amplitudes and sensory conduction velocities (SCV) are normal in sural and left superficial fibular nerves.
Presence of bilateral sural sparing.

Appendix Table 3. Blink Reflex*

Trial	NR	R1 (ms)	R2i (ms)	R2c (ms)	R2i - R2c (ms)
Left	1.8	22.8	45.3	45.6	0.30
Right		27.2	51.3	44.5	6.80
L-R		4.4	6.0	1.1	6.50

*Blink reflex with early ipsilateral responses (R1) showing markedly increased latencies, and late ipsilateral (R2i) and contralateral (R2c) responses with mild/slightly increased latencies.

Appendix Table 4. F Waves*

Nerve (Muscle)	F-M Lat (ms)	F/M Ratio	L-R Mean-F	Mean-F	Persistence	Dispersion	Max-F	Min-F
Left Fibular (m. EDB)	51.09	1.46	9.80	59.30	100.00	1.87	60.23	58.36
Right Fibular (m. EDB)	62.66	1.37	9.80	69.10	40.00	0.63	69.38	68.75
Left Median (m. APB)	33.13	6.27	4.53	45.75	100.00	1.10	46.41	45.31
Right Median (m. APB)	33.44	12.15	4.53	50.28	100.00	11.25	56.72	45.47
Left Tibial (m. AbdH)	35.16	3.34	5.18	48.46	20.00	2.04	49.38	47.34
Right Tibial (m. AbdH)	35.16	4.33	5.18	43.28	20.00	0.31	43.44	43.13
Left Ulnar (m. hypothenar)	39.49	3.21	8.35	44.68	57.14	0.82	43.44	44.18
Right Ulnar (m. hypothenar)	28.95	2.85	8.35	36.33	70.00	4.34	37.97	33.63

*F-waves with normal mean latencies in tibial nerves and mild (left fibular) to moderate/severe increased latencies (right fibular and median nerves) in others (right ulnar, left fibular, and right fibular nerves).

Appendix Table 5. H Reflex – Upper and Lower Limbs*

Nerve	H-Lat (ms)	Max H-Amp (mV)	L–R H-Lat (ms)	M-Lat (ms)	M-Amp (mV)	H–M Lat (ms)	H/M Ratio
Left Tibial L5, S1 *(m. gastrocnemius)*	absent	—	—	6.72	3.17	—	—
Right Tibial L5, S1 *(m. gastrocnemius)*	absent	—	—	6.72	6.41	—	—

*H Reflex (L5–S1) absent in lower limbs (LLs).
